# Effects of Deforestation and Fragmentation on Tree Beta Diversity and Endemism Across Landscapes in the Atlantic Forest Biodiversity Hotspot

**DOI:** 10.1111/gcb.70855

**Published:** 2026-04-09

**Authors:** Jean M. Freitag Kramer, Victor P. Zwiener, Mateus Camana, Renato A. F. de Lima, Sandra Cristina Müller

**Affiliations:** ^1^ Laboratório de Ecologia Vegetal (LEVEG), Departamento de Ecologia Universidade Federal do Rio do Sul (UFRGS) Porto Alegre Brazil; ^2^ Laboratório de Ecologia e Biogeografia de Plantas, Departamento de Biodiversidade, Setor Palotina Universidade Federal do Paraná (UFPR) Palotina Brazil; ^3^ Programa de Pós‐Graduação em Ecologia Universidade Federal do Rio do Sul (UFRGS) Porto Alegre Brazil; ^4^ Departamento de Ciências Biológicas, ESALQ Universidade de São Paulo São Paulo Brazil

**Keywords:** biodiversity change, biodiversity loss, biotic homogenization, community composition, habitat fragmentation, tropical forest

## Abstract

Biodiversity is highly heterogeneous across space, shaped by factors such as climate, geographical distance, and human activities. However, as we enter the Anthropocene, the impacts of habitat loss and fragmentation on biodiversity are becoming increasingly severe and insufficiently disentangled. Here we assess how habitat amount, habitat fragmentation, regional climate, and geographical distance influence beta diversity and endemism levels in tree communities across 95 landscapes in the Brazilian Atlantic Forest. We generated landscapes of 100 km^2^ around forest inventory data extracted from the Neotropical Tree Communities database (TreeCo). Each landscape was characterized in terms of habitat amount, fragmentation (number of patches, mean patch size, and mean distance to the nearest neighbor), mean spatial distance (geographical distance among forest inventories), and climate across the region. The landscape variables (habitat amount and fragmentation) and the mean spatial distance were stronger predictors of beta diversity and endemism level when compared to regional climate. Beta diversity declined with increasing fragmentation, suggesting biotic homogenization in highly fragmented landscapes. On the other hand, spatial distance promoted increases in beta diversity, with more dissimilar communities as the distance increased. Moreover, endemism increased with habitat amount, with higher endemism levels in landscapes with more habitat. We highlight that our study contributes to the ongoing debate on the effects of fragmentation on biodiversity, reporting negative effects of habitat fragmentation on beta diversity within landscapes across the entire Atlantic Forest. Our findings successfully separate the effects of habitat fragmentation, habitat amount, and spatial distance on biodiversity, providing a better understanding of the processes that determine biodiversity change at the landscape scale. We advocate for conservation strategies that simultaneously protect both large and smaller habitat patches to sustain tree beta diversity and endemism across human‐modified landscapes.

## Graphical abstract

Habitat loss and fragmentation reshape biodiversity patterns around the world. Across 95 landscapes in the Brazilian Atlantic Forest, we show that habitat amount, fragmentation, and spatial distance are stronger drivers of tree beta diversity and endemism than regional climate. Habitat fragmentation promotes biotic homogenization (reduced beta diversity), while greater habitat amount and spatial distance sustain higher endemism and beta diversity. Protecting both large and small forest patches is essential to maintain biodiversity in human‐modified landscapes.

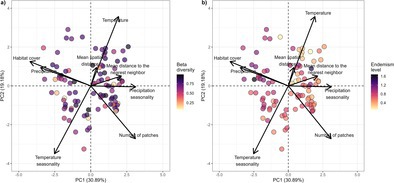

## 1 Introduction

Biodiversity is highly heterogeneous across the Earth and different factors influence its distribution, including climate, geographical distance, and human activities (Kraft et al. 2011; Pimm et al. 2014; Coelho et al. 2023). As we enter the Anthropocene, the impacts of human activities on biodiversity, such as habitat loss and habitat fragmentation, are becoming increasingly severe (Haddad et al. 2015; Taubert et al. 2018; Hansen et al. 2020). Thus, habitat loss and fragmentation have emerged as key drivers of biodiversity change and loss worldwide (Taubert et al. 2018; Chase et al. 2020; Zhang et al. 2024).

Habitat loss (i.e., reduction of habitat area and consequently the available amount of habitat) and habitat fragmentation (i.e., division of the habitat into several parts and represents a change in habitat configuration) are distinct processes (Fahrig 2003; Fischer and Lindenmayer 2007). While these processes are separate (Fahrig 2003), they frequently occur together, complicating efforts to disentangle the specific impacts of each (Palmeirim et al. 2019; Püttker et al. 2020; Riva et al. 2024). Therefore, it is essential to measure both processes at the landscape scale, capturing differences in patch configuration across landscapes while accounting for habitat amount to effectively distinguish the effects of habitat loss and fragmentation on biodiversity and ecosystem functions (McGarigal and Cushman 2002; Riva et al. 2024; Fahrig 2017; Fahrig et al. 2026).

Evidence suggests that habitat loss has stronger negative impacts on biodiversity than habitat fragmentation (Palmeirim et al. 2019; Fahrig et al. 2019). Yet the effects of habitat fragmentation per se (i.e., the division of habitat into multiple patches for a given habitat amount—sensu Fahrig 2017) remain debated. While many studies report neutral or even positive effects of fragmentation on biodiversity (Fahrig et al. 2019; Palmeirim et al. 2019; Galán‐Acedo et al. 2024; Riva et al. 2024), other studies have observed negative impacts (Fletcher et al. 2018; Püttker et al. 2020), leaving the overall influence of fragmentation on biodiversity (whether negative, neutral, or positive) uncertain and dependent of the context. In addition, most of these studies have focused on the impacts of fragmentation per se (hereafter referred to as habitat fragmentation) on alpha diversity, especially species richness (Fahrig 2017; Fahrig et al. 2019), while its effects on other dimensions of biodiversity, such as beta diversity, remain less understood.

Habitat loss and fragmentation can change community composition, thereby affecting beta diversity, and driving the processes of biotic homogenization and differentiation (Socolar et al. 2016; Kramer, Zwiener, et al. 2023; Blowes et al. 2024; Maurenza et al. 2024). Evidence shows that habitat loss and fragmentation can either increase or decrease beta diversity in human‐modified landscapes (Arroyo‐Rodríguez et al. 2013; Solar et al. 2015; Collins et al. 2017; Barreto et al. 2023), with both biotic homogenization and differentiation potentially occurring (Socolar et al. 2016; Kramer, Zwiener, et al. 2023; Kramer, Bald, et al. 2023). However, most of the available studies on the effects of habitat loss and fragmentation have focused on a patch‐to‐landscape scale, typically examining a limited range of habitat cover (e.g., ~5% to 50%) (Püttker et al. 2015; Barreto et al. 2023). Only a few studies have extended this approach across the full gradient of habitat cover (e.g., ~0% to ~100%) to assess the effects of habitat fragmentation on alpha diversity (Palmeirim et al. 2019; Püttker et al. 2020). Therefore, the effects of habitat fragmentation on beta diversity across a complete gradient of habitat amount remain largely unexplored.

The Atlantic Forest represents a natural laboratory for evaluating the effects of habitat loss and fragmentation on beta diversity, given its high biodiversity and extensive human impacts (Myers et al. 2000; de Lima, Souza, et al. 2020; de Lima, de Oliveira, et al. 2020). The Atlantic Forest exhibits high environmental heterogeneity and a pronounced gradient of habitat amount and fragmentation over the 28% of native forest cover that remains from the original coverage (Ribeiro et al. 2009; Rezende et al. 2018). Current forest coverage ranges from large continuous forest areas to landscapes dominated by small and isolated forest fragments, where > 90% of these have < 50 ha (Ribeiro et al. 2009; Vancine et al. 2024). Given this scenario of a highly fragmented biome and the growing recognition of the importance of small forest fragments for biodiversity conservation (Wintle et al. 2019; Riva and Fahrig 2022), further investigation of fragmentation effects on beta diversity is crucial for the conservation of species‐rich tropical biodiversity hotspots, such as the Atlantic Forest.

Here we aim to evaluate how habitat amount and fragmentation influence tree beta diversity and endemism levels across a gradient of habitat amount in 95 landscapes in the Brazilian Atlantic Forest. Using a landscape‐scale approach, we assess the effects of habitat amount and fragmentation after controlling for the potential influence of regional climate and geographical distance on beta diversity and endemism (Zwiener et al. 2018, 2020; de Lima, de Oliveira, et al. 2020; Klipel et al. 2022). Given that both biotic homogenization and differentiation processes can potentially occur in human‐modified landscapes (Socolar et al. 2016; Kramer, Zwiener, et al. 2023), two contrasting patterns are anticipated: (1) a higher beta diversity in highly fragmented landscapes with low habitat amount, suggesting recent biotic differentiation; and (2) a lower beta diversity in similarly highly fragmented, low‐habitat landscapes, indicating long‐term or ongoing biotic homogenization. Moreover, we predict that less fragmented landscapes with higher habitat amounts will support greater endemism levels of tree species.

## 2 Material and Methods

### 2.1 Forest Inventories

We used forest inventory data stored in the Neotropical Tree Communities database (TreeCo) (de Lima et al. 2015; de Lima, de Oliveira, et al. 2020), selecting forest inventories (communities) conducted within the limits of the Atlantic Forest (SOS Mata Atlântica and INPE 2018; Muylaert et al. 2018). To minimize possible noises and sampling inconsistencies among forest inventories, we applied three filters to the TreeCo database to obtain the forest inventories: (1) a minimum sampling effort of 0.10 ha (ha) to mitigate biases from small sample sizes; (2) a diameter at breast height (DBH) cutoff of ≥ 5 cm to exclude juvenile trees and shrubs; and (3) complete data on species identification and abundance, to ensure accurate calculation of beta diversity and endemism at the species level.

### 2.2 Landscape Units

**FIGURE 1:**
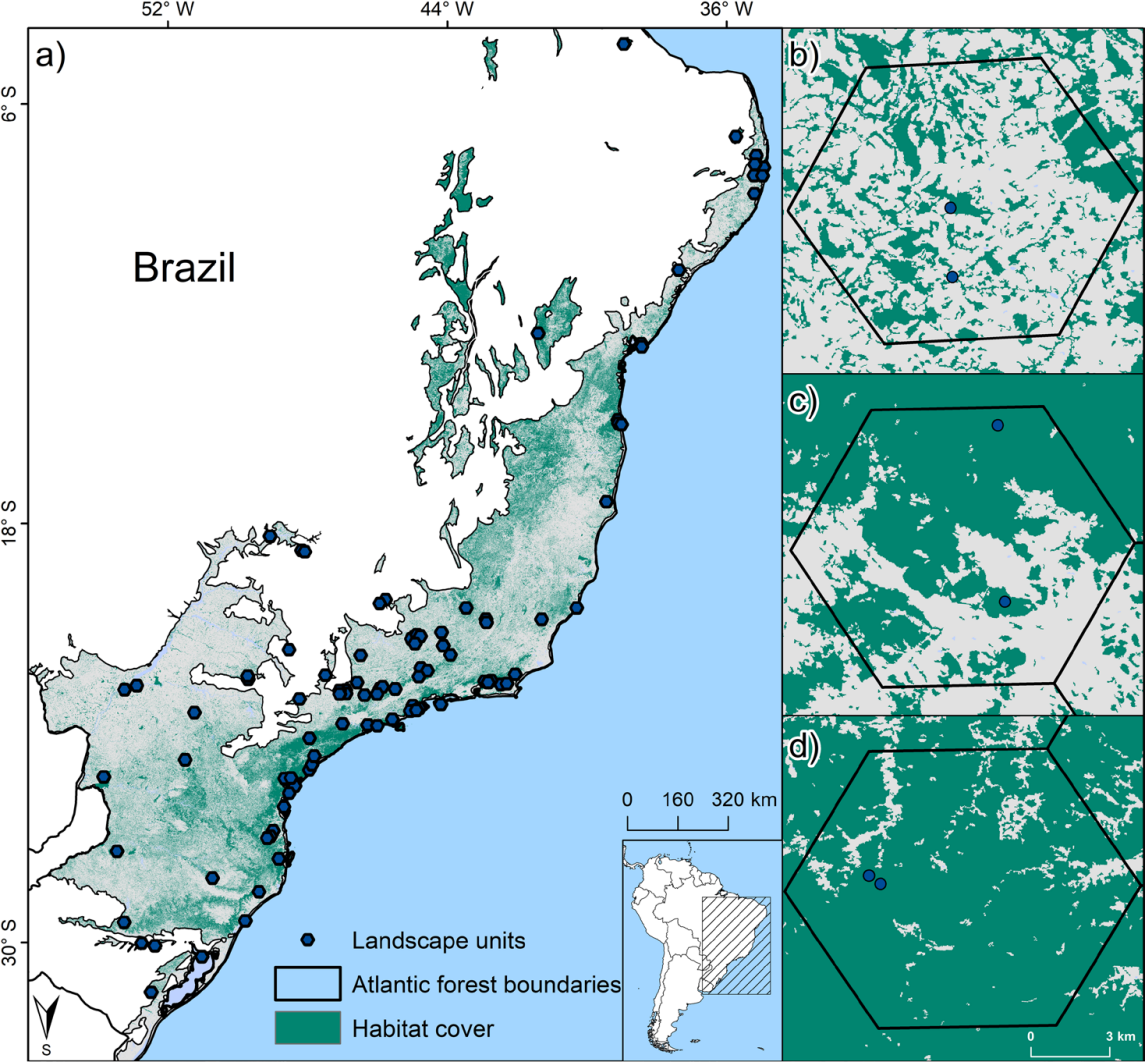
Spatial distribution of the 95 studied landscapes (dark points) of 100 km2 across the Brazilian Atlantic Forest (a). The shaded area represents the original extension of the Atlantic Forest domain in Brazil, whereas the current forest vegetation cover (i.e., habitat cover) is exhibited in green. On the right, we are showing three examples of landscapes with distinct levels of habitat amount and fragmentation. The landscape in panel (b) presents a low level of habitat amount and a high level of habitat fragmentation; the landscape in panel (c) presents intermediate levels of both habitat amount and fragmentation; and the landscape in panel (d) presents high levels of habitat amount and a low level of fragmentation.

After selecting the forest inventories from Neotropical Tree Communities database (TreeCo), we generated a grid of hexagon cells of 100 km^2^ to serve as our landscape sampling units (hereafter referred to as landscape). Hexagonal cells were chosen because they are better suited to represent the landscape structure and their spatial configurations when compared with rectangular cells (Birch et al. 2007; Barreto et al. 2010). This landscape size of 100 km^2^ is commonly used to characterize regional landscapes affecting local communities (in our case, forest inventories) (Pardini et al. 2010; Camargo et al. 2018; Palmeirim et al. 2019). As we were interested in beta diversity (i.e., compositional dissimilarity among communities), we selected landscapes with at least two forest inventories for data extracting and analysis, totaling 95 landscapes composed of 270 forest inventories along the Brazilian Atlantic Forest (Figure 1). The mean number of forest inventories per landscape was 2.84, ranging from 2 to 12 forest inventories (Appendix S1).

### 2.3 Landscape Variables

For each landscape, habitat amount and habitat fragmentation were measured based on land cover maps at 30‐m spatial resolution retrieved from Project MapBiomas collection 8.0 (https://mapbiomas.org) (Souza et al. 2020). The year of extraction was based on the oldest year among the forest inventories within each landscape, which allowed to ensure that the amount of habitat and its spatial configuration (habitat fragmentation) matched the time when data were collected for each landscape. The definition of native habitat includes the forest vegetation types according to MapBiomas (Souza et al. 2020) and was based on the classes: Forest Formation, Savanna Formation, Mangrove, Floodable Forest and Wooded Sandbank Vegetation. These classes represent the different native formations that occur in the Atlantic Forest, including dense, open, and mixed ombrophilous forest, semideciduous and deciduous seasonal forest, and pioneer formations. All the remaining classes of land cover (e.g., native grasslands) and land use (e.g., agriculture and urban) were considered as non‐habitat (no data).

Within each landscape, habitat amount was measured as the percentage of remaining native habitat cover (% of habitat cover), and habitat fragmentation was measured by the number of patches, mean patch size and mean distance to the nearest neighbor. We choose number of patches, mean patch size and mean distance to the nearest neighbor as our fragmentations metrics because they are directly linked to the definition of habitat fragmentation and represent the main effects of habitat fragmentation, resulting in an increase in the number of patches, decrease in patch sizes, and increase in isolation among patches (mean distance to the nearest neighbor) in the landscape (McGarigal and Cushman 2002; Fahrig 2003). In addition, we also extracted the mean spatial distance calculated as the geographical distance (kilometers) among forest inventories within each landscape, which may influence beta diversity (König et al. 2017; Graco‐Roza et al. 2022). Habitat cover and mean spatial distance were extracted using QGIS (QGIS Development Team 2024), whereas the number of patches and mean patch size were calculated using *raster* package, and mean distance to the nearest neighbor using function “lsm_c_enn_mn” in *landscapemetrics* package in R environment (Hesselbarth et al. 2019; R Core Team 2023; Hijmans et al. 2024).

### 2.4 Climate Variables

To represent the regional climate variation, we extracted within each landscape the following variables: annual mean temperature (°C), temperature seasonality (°C), total annual precipitation (mm), and precipitation seasonality (%). We chose these variables because they have been reported as important drivers affecting plant community composition in the Brazilian Atlantic Forest (Marcilio‐Silva et al. 2017), as well as in others tropical forests globally (McFadden et al. 2019; He et al. 2020; Qian et al. 2020, 2024). Furthermore, these climatic variables represent the primary ecological axes of environmental space (König et al. 2017; He et al. 2020). The climate variables were obtained from the WorldClim database at 1‐km resolution, considering monthly averages from 1970 to 2000 (Fick and Hijmans 2017; http://www.worldclim.org) and extracted for each individual landscape using QGIS (QGIS Development Team 2024).

### 2.5 Estimation of Beta Diversity

The beta diversity within each landscape was computed based on species abundances using two approaches. First, beta diversity was estimated based on the Bray–Curtis dissimilarity index using the total dissimilarity derived from two components: balanced changes in abundance (turnover) and abundance gradients (nestedness) (Baselga 2017). This measure represents the overall variation in community composition in each landscape (hereafter called total beta diversity). Second, because standard beta diversity indices, such as Bray‐Curtis and Sørensen, are influenced by alpha diversity patterns (Chase et al. 2011), we used the modified Raup‐Crick beta diversity index (Raup and Crick 1979), which accounts for variation in alpha diversity among sampling sites. The Raup‐Crick beta diversity tests the probability of two communities being more or less dissimilar compared to a null expectation considering the number of species (alpha diversity) present in each community (Chase et al. 2011; Stegen et al. 2013). The Raup‐Crick beta diversity index naturally ranges from −1 to +1 and indicates whether community composition is more similar (close to −1) or more dissimilar (close to +1) than expected by chance. Moreover, values close to −1 and +1 reflect that communities are governed by determinist processes, whereas values close to 0 indicate that species variation in the communities is governed by stochastic processes (Chase et al. 2011; Stegen et al. 2013; Barreto et al. 2023). However, we rescaled the Raup‐Crick beta diversity to range from 0 to 1 to enable direct comparisons with total beta diversity. Total beta diversity was calculated using the function “beta.multi.abund” in the *betapart* package (Baselga et al. 2023) and Raup‐Crick beta diversity using the function “RC.pc” in the *iCAMP* package in R (Ning et al. 2020; Ning 2022; R Core Team 2023).

### 2.6 Endemism Levels

Species endemism levels were obtained for each community based on species occurrences in different continents, countries, and Brazilian states (Oliveira‐Filho 2010; Filardi et al. 2018; de Lima, de Oliveira, et al. 2020). We used the endemism classification following de Lima, de Oliveira, et al. (2020), where for each species, endemism was categorized as follows: (i) exotic—introduced or naturalized species; (ii) not endemic—species with Pantropical, Neotropical, and South American distributions; (iii) regional endemic—species restricted to South, South‐eastern or Northeastern regions in Brazil; and (iv) local endemic—species restricted to one or two adjacent regions in Brazil (e.g., São Paulo and Rio de Janeiro states). For each level of species endemism was attributed a score: exotic = −1, not endemic with Pantropical and Neotropical distribution = 0, not endemic with South American distribution = 1, regional endemic = 2, and local endemic = 3. We considered that the presence of exotic species has a negative score on endemism level and as higher the endemism level, higher its score. Then, the endemism level was measured using the community‐weighted mean (CWM) (Lavorel et al. 2008) based on species abundance, and as higher the value of CWM, higher the endemism level of that community. The CWM was calculated using the function “functcomp” in *FD* package in R (Laliberté et al. 2023; R Core Team 2023). To obtain a value of endemism level for each landscape, we calculated the average of the CWM values for all communities within the landscape.

### 2.7 Data Analysis

To reduce possible correlations among the variables, we assessed Pearson coefficient of correlation (*r*) and visualized the relationships using scatterplots, which is useful to detect observations that do not comply with the general pattern between two variables (Zuur et al. 2010). After that, we retained only the variables considered as not highly correlated (i.e., those with *r* ≤ 0.70) (Dormann et al. 2013). Across all 95 landscapes, habitat cover was highly correlated with mean patch size (*r* = 0.94; Appendix S2). To avoid possible bias in the results of the models due to highly correlated variables (Dormann et al. 2013; Ruffell et al. 2016), we removed mean patch size from the models and kept habitat cover as our habitat amount metric and number of patches together with mean distance to the nearest neighbor as our habitat fragmentation metrics. In addition, the four climate variables, annual mean temperature, temperature seasonality, annual precipitation, and precipitation seasonality, were not highly correlated (*r* < 0.58; Appendix S3) and were thus maintained in the analyses. Moreover, total beta diversity, Raup‐Crick beta diversity, and endemism level were also not highly correlated (*r* < 0.53; Appendix S4) and were maintained. The Pearson correlation coefficients and scatterplots comparing the relationships between variables are exhibited in Appendices S2–S4.

Firstly, to explore the overall variation on the 95 landscapes described by landscape variables (habitat cover, number of patches and mean distance to the nearest neighbor), mean spatial distance between forest inventories within each landscape, and regional climate (temperature, temperature seasonality, precipitation and precipitation seasonality) in relation to beta diversity and endemism levels, we performed a principal component analyses (PCAs) using the “prcomp” function in *stats* R base package (Venables and Ripley 2002; R Core Team 2023).

To effectively separate the effects of habitat fragmentation from those of habitat amount, there must be no correlation between the fragmentation metrics and habitat amount across the sampled landscapes. One solution is to control statistically for any association between these variables, either by including an interaction term or by incorporating habitat amount directly into the models (Wang et al. 2014; Ruffell et al. 2016; Riva et al. 2024). Considering this, we ran models with the fragmentation metrics alone, models with habitat amount alone, and models including the interaction between the two.

Secondly, to assess the effects of landscape variables (habitat cover, number of patches and mean distance to the nearest neighbor), mean spatial distance between forest inventories within each landscape, and regional climate variables (temperature, temperature seasonality, precipitation and precipitation seasonality) on beta diversity and endemism level within each landscape, we used generalized linear models (GLM). The landscape, mean spatial distance, and climate variables were considered as predictor variables, whereas the total beta diversity, Raup‐Crick beta diversity, and endemism level were considered as response variables in the models. We performed full models with each set of predictor variables (landscape, mean spatial distance, and climate) for each response variable (total beta diversity, Raup‐Crick beta diversity and endemism level), including an interaction term between habitat cover and number of patches. After running a full model, we tested all possible combinations of predictors for each response variable to generate subsets of the global model via the function “dredge” in *MuMIn* package (Barton 2024). The function “dredge” performs automated model selection by generating subsets of the supplied global model resulting in a set of models with “all possible” combinations (Burnham and Anderson 2002; Barton 2024). The models were ranked based on their Akaike Information Criteria (AIC) and a model selection procedure was performed to select the best models, considering those with ΔAIC ≤ 2 as the best and most parsimonious (Burnham and Anderson 2002). This approach allows us to identify which are the best predictors regarding landscape, mean spatial distance, and climate variables that affect beta diversity and endemism levels of tree communities across landscapes in the Brazilian Atlantic Forest.

Appropriate error distributions were used for each response variable in the models. Total beta diversity and Raup‐Crick beta diversity ranged between zero and one and did not follow a normal distribution (*p* value < 0.05 regarding Shapiro–Wilk test of normality), so the beta distribution was applied to these variables (Cribari‐Neto and Zeileis 2010), a distribution that easily accommodates asymmetries and is appropriate for response variables varying from zero to one (Cribari‐Neto and Zeileis 2010). The endemism level followed a normal distribution (*p* value > 0.05 regarding Shapiro–Wilk test of normality), so a gaussian distribution was applied for these variables in the models. Beta regression was performed using function “betareg” in *betareg* package (Zeileis et al. 2022) and linear regression with gaussian distribution was performed using function “lm” in *stats* R base package (R Core Team 2023).

The predictors variables were standardized to zero mean and unit variance to establish a meaningful comparison between them (Schielzeth 2010) using the function “decostand” in *vegan* package (Oksanen et al. 2022). To evaluate spatial autocorrelation, we applied Moran's *I* on model residuals (Dormann et al. 2007) for each of the best selected models following AIC and they did not present spatial autocorrelation (Moran's *I* test *p* value > 0.05). Spatial autocorrelation was estimated using *ape* package (Paradis et al. 2024). In addition, the multicollinearity of the predictors variables within the selected models was assessed by calculating the variance inflation factor (VIF). As VIF values of the selected models were < 3, indicating they were not highly collinear, no additional variable exclusion was needed (Zuur et al. 2010). VIF was calculated using “vif” function in *car* package (Fox et al. 2023).

All analyses were conducted in R environment version 4.2.3 (R Core Team 2023) and the *ggplot2* package was used for graphical plotting (Wickham 2016).

## 3 Results

**FIGURE 2:**
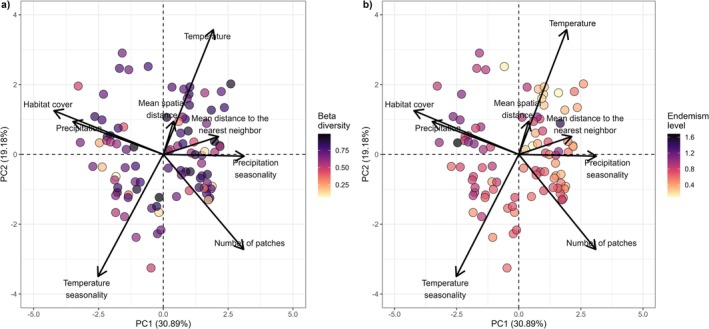
Representation of the first two axes of the principal component analysis (PCA) characterizing the environmental space across the 95 studied landscapes in the Brazilian Atlantic Forest. Arrows indicate the loadings of each variable on the first two PCA axes (PC1 and PC2), which together explain 50.07% of the total variation in the environmental space. The points represent the landscapes, which are colored according to their total beta diversity (a) and endemism level (b). Warmer colors indicate lower values and colder colors higher values of beta diversity and endemism.

The 95 studied landscapes represent an entire gradient of habitat amount along the Brazilian Atlantic Forest, ranging from ~0% to ~100% of habitat cover (Appendix S5). The first two axes of PCAs captured 50.07% of the variation across the landscapes being described by landscape variables (habitat cover, number of patches, and mean distance to the nearest neighbor), mean spatial distance, and regional climate variables (temperature, temperature seasonality, precipitation, and precipitation seasonality) (Figure 2). Landscapes with higher beta diversity (Figure 2a) are not strongly clustered in specific regions of the ordination space, suggesting that beta diversity is not tightly linked to the dominant gradients captured by the first two PCA axes (Appendix S6). In contrast, endemism levels (Figure 2b) show a clearer pattern along the ordination space, with higher endemism in landscapes that combine higher habitat cover and precipitation (Appendix S6).

We found different sets of models influencing beta diversity and endemism level of tree communities across landscapes in the Brazilian Atlantic Forest, with some variables influencing positively and others negatively. The landscape variables (specifically habitat cover and number of patches) and the mean spatial distance were the strongest predictors compared to regional climate. The best selected models did not include interaction between habitat amount and habitat fragmentation.

### 3.1 Beta Diversity

**FIGURE 3:**
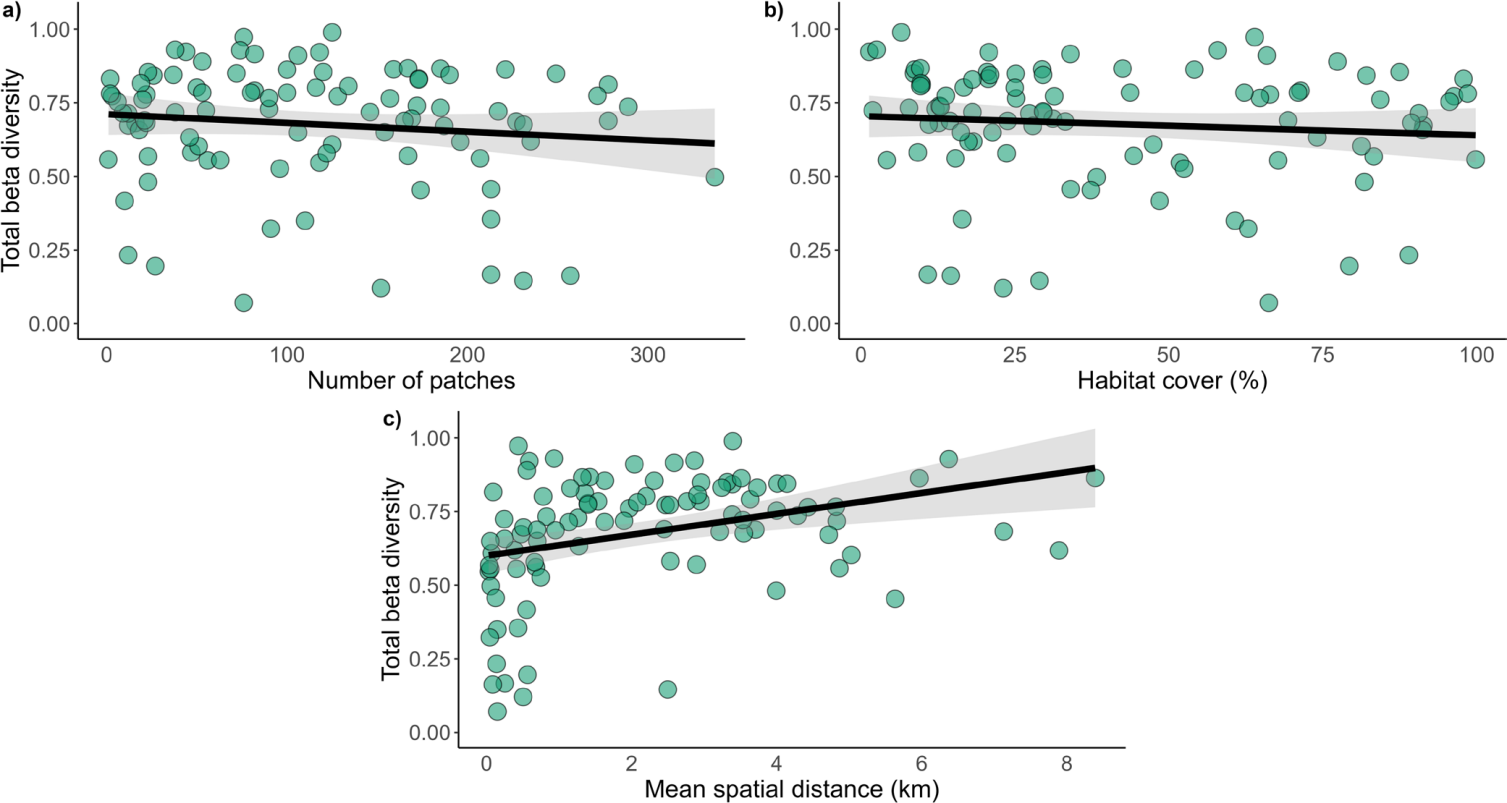
The strongest variables affecting total beta diversity. The number of patches (a) and habitat cover (b) affects total beta diversity negatively, whereas the mean spatial distance (c) between forest communities within each landscape affects positively. Solid lines represent the relationships between variables and shaded gray areas represent the confidence intervals. The green points represent the landscapes along the Brazilian Atlantic Forest.

For total beta diversity, the selected models include the effect of mean spatial distance, number of patches, and habitat cover as the strongest predictors (Table 1). Mean spatial distance had the strongest effect, positively affecting total beta diversity (standardized effect size = 0.30), followed by number of patches (standardized effect size = −0.26) and habitat cover (standardized effect size = −0.26), which negatively affected total beta diversity (Figure 3). That is, landscapes with more habitat patches (more fragmented) and more habitat amount had lower beta diversity. The mean distance to the nearest neighbor and regional climate variables did not significantly influence total beta diversity (*p* > 0.05).

**TABLE 1:** Best beta regression models for total beta diversity following AIC statistics.

Models	AIC	ΔAIC	*R* ^2^	Explanatory variables	Effect	*p*
Model 1	−57.80	0	0.16	Mean spatial distance	0.30	0.0004*
				Number of patches	−0.26	0.01*
				Habitat cover	−0.26	0.01*
Model 2	−57.39	0.40	0.18	Mean spatial distance	0.29	0.0009*
				Number of patches	−0.30	0.005*
				Habitat cover	−0.25	0.01*
				Precipitation seasonality	0.12	0.17
Model 3	−56.46	1.33	0.17	Mean spatial distance	0.31	0.0003*
				Number of patches	−0.31	0.006*
				Habitat cover	−0.31	0.007*
				Temperature	−0.08	0.33
Model 4	−55.82	1.97	0.16	Mean spatial distance	0.31	0.0003*
				Number of patches	−0.27	0.01*
				Habitat cover	−0.29	0.01*
				Precipitation	0.06	0.55
Model 5	−55.82	1.97	0.18	Mean spatial distance	0.29	0.0007*
				Number of patches	−0.34	0.003*
				Habitat cover	−0.30	0.01*
				Precipitation seasonality	0.11	0.19
				Temperature	−0.07	0.39

*Note:* All models with ΔAIC ≤ 2 are exhibited and ranked based on their AIC. Asterisks indicate variables that had significant effects (*p* < 0.05).

Abbreviations: ΔAIC, AIC difference from the best model; AIC, Akaike information criteria; Effect, standardized effect size; explanatory variables, predictors variables included in the model; Models, models name; *p*, significance *p* value; *R* ^2^, explained variance by the model.

When calculating beta diversity controlling for the effects of alpha diversity (i.e., Raup‐Crick beta diversity), the selected models include only the effect of mean spatial distance as the strongest (standardized effect size = 0.31) and significant predictor (*p* < 0.05) (Appendices S7 and S8). This means that what affects beta diversity independent of changes in alpha diversity in our landscapes is the mean spatial distance between forest inventories within each landscape. Habitat cover, number of patches, mean distance to the nearest neighbor and regional climate variables did not influence significantly Raup‐Crick beta diversity (*p* > 0.05). Moreover, most Raup‐Crick beta diversity values are positive and distant from zero, indicating higher values than expected by chance given the observed alpha diversity.

### 3.2 Endemism Level

**FIGURE 4:**
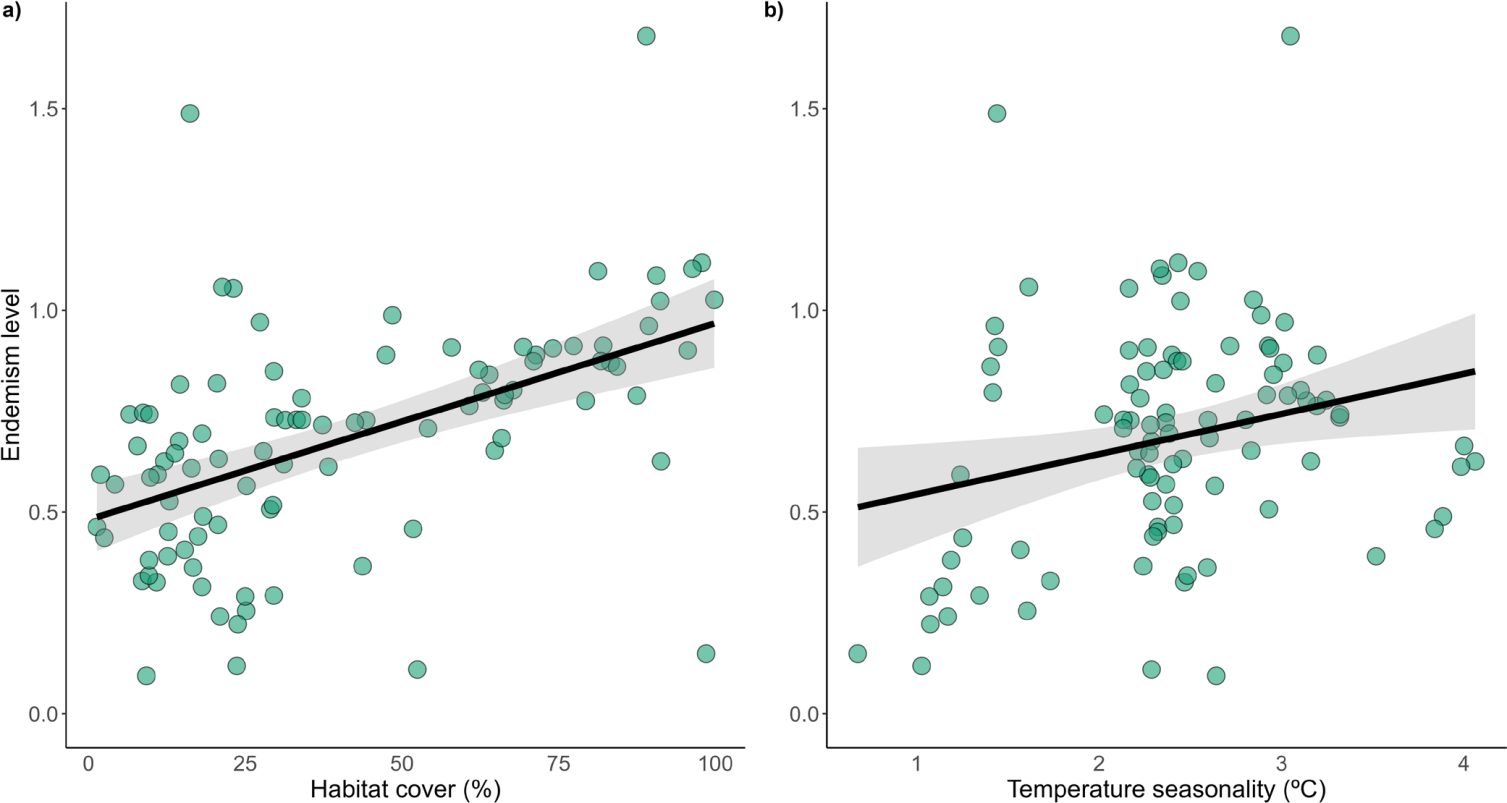
The strongest variables affecting endemism level. Habitat cover (a) and temperature seasonality (b) positively affect the endemism level. Solid lines represent the relationships between variables and shaded gray areas represent the confidence intervals. The green points represent the landscapes along the Brazilian Atlantic Forest.

Habitat cover (standardized effect size = 0.13) and temperature seasonality (standardized effect size = 0.05) were the strongest predictors, affecting positively endemism level (Figure 4). This means that landscapes with more habitat availability (i.e., habitat amount) and more temperature seasonality had higher contributions of endemic species in the sampled communities. The remaining variables did not significantly affect the endemism level (*p* > 0.05) (Table 2).

**TABLE 2:** Best linear regression models for endemism level following AIC statistics.

Models	AIC	ΔAIC	*R* ^2^	Explanatory variables	Effect	*p*
Model 1	3.43	0	0.28	Habitat cover	0.13	0.0002*
				Temperature seasonality	0.05	0.03*
Model 2	5.09	1.65	0.27	Habitat cover	0.13	0.0002*
				Temperature seasonality	0.05	0.04*
				Mean spatial distance	−0.01	0.46
Model 3	5.30	1.86	0.27	Habitat cover	0.12	0.0004*
				Temperature seasonality	0.05	0.03*
				Precipitation	0.01	0.55

*Note:* All models with ΔAIC ≤ 2 are exhibited and ranked based on their AIC. Asterisks indicate variables that had significant effects (*p* < 0.05).

Abbreviations: ΔAIC, AIC difference from the best model; AIC, Akaike information criteria; Effect, standardized effect size; explanatory variables, predictors variables included in the model; Models, models name; *p*, significance *p* value; *R* ^2^, explained variance by the model.

## 4 Discussion

Our results suggest that landscape variables and spatial distance are the main drivers of change in tree beta diversity and endemism level in the Brazilian Atlantic Forest. Differently from previous studies that report positive effects of fragmentation (e.g., Fahrig et al. 2019; Galán‐Acedo et al. 2024), we observed negative effects of habitat fragmentation on beta diversity, where an increase in fragmentation was associated with lower beta diversity (Gonçalves‐Souza et al. 2025). Considering this and given that the distance between communities within the landscape positively influenced beta diversity, our results suggest an ongoing process of biotic homogenization occurring in tree communities in highly fragmented landscapes in the Atlantic Forest. Finally, as we predicted, a high endemism level was observed in landscapes with high amounts of habitat, emphasizing the importance of habitat conservation for maintaining biodiversity.

In recent decades, different studies have investigated the role of spatial and climatic factors that are responsible for variation in beta diversity of plant communities (e.g., König et al. 2017; He et al. 2020; Zwiener et al. 2020; Leo et al. 2024; Qian et al. 2024). Interestingly, our findings report a stronger effect of landscape variables and spatial distance in comparison with regional climate on beta diversity. As here we evaluated how habitat amount and fragmentation influence tree beta diversity and endemism at a landscape (i.e., regional) scale, the regional climate variables within each landscape acted as a control to represent the climate variation across the Atlantic Forest. Previous studies also conducted at a landscape scale did not account for climate variation within the examined landscapes (e.g., Palmeirim et al. 2019; Püttker et al. 2020; Gonçalves‐Souza et al. 2025). This is a differential of our study, and on the scale that we evaluated the communities (i.e., landscapes of 100 km^2^), we can be confident that the effects of landscape variables and spatial distance stand out in comparison with regional climate.

Our results indicate a positive effect of the spatial distance on beta diversity. Thus, as the geographical distance increases between forest communities within each landscape, beta diversity also increases, supporting the well‐established concept of distance decay of similarity (or distance increase of dissimilarity) (Nekola and White 1999; Soininen et al. 2007; Graco‐Roza et al. 2022). The distance decay pattern in community composition occurs because species dispersal potential declines considerably with greater distances, which can be due to dispersal barriers, such as isolated forest patches, which limit dispersion, or from neutral processes, where propagule pressure is lower at greater distances than in nearby areas (Nekola and White 1999; Soininen et al. 2007; Buhk et al. 2017). However, most of these studies detect a pattern of distance decay considering broader geographical scales, ranging in thousands of kilometers (e.g., Soininen et al. 2007; König et al. 2017; Graco‐Roza et al. 2022), and our study was one of the few that detect this pattern at a landscape scale (but see Buhk et al. 2017). We observed an initial increase followed by a slight stabilization in beta diversity within distances of < 2 km in our landscapes. This pattern is potentially associated with the high proportion of rare (low‐density) species in tree communities in tropical forests (Hordijk et al. 2024; de Lima et al. 2024). Additionally, tropical tree species usually have a pattern of aggregated distribution (Condit et al. 2000; Wiegand et al. 2025), further contributing to this pattern of dispersal limitation for tree communities even within a landscape scale in the Brazilian Atlantic Forest. In addition, by separating the effects of habitat fragmentation and spatial distance on beta diversity, we better understood how these processes affect biodiversity change.

Contrary to studies suggesting that habitat fragmentation increases beta diversity at a patch‐to‐landscape scale (e.g., Arroyo‐Rodríguez et al. 2013; Sfair et al. 2016; Fahrig et al. 2022), our results show a negative relationship between habitat fragmentation (measured by the number of patches) and beta diversity, with lower values of beta diversity observed in more fragmented landscapes. This decline in beta diversity as the number of fragments increases in a given landscape area may result from the proliferation and persistence of widespread generalist species in these highly fragmented landscapes (Lôbo et al. 2011; Gonçalves‐Souza et al. 2025). Habitat fragmentation not only increases the number of patches, reduces the mean patch size, and increases the amount of edge of the fragments (Fahrig 2003; Fischer and Lindenmayer 2007), but may also reduce habitat quality within fragments in the landscape (Mortelliti et al. 2010; Vicente et al. 2024), which facilitates the proliferation of widespread generalist species and may result in biotic homogenization (Lôbo et al. 2011; Tabarelli et al. 2012). Fragmented landscapes dominated by small and isolated patches are more exposed to anthropogenic disturbances (e.g., cattle intrusion), which can degrade habitat quality and disproportionately affect specialist species while favoring widespread generalist species (Faria et al. 2023; Vicente et al. 2024). Thus, the configuration of fragmented landscapes may facilitate not only spatial isolation but also internal disturbances that degrade habitat quality, reinforcing compositional shifts toward the proliferation of widespread generalist species (Faria et al. 2023; Vicente et al. 2024). This interaction between landscape fragmentation and local habitat degradation likely acts synergistically and may contribute to the observed patterns of biotic homogenization. While previous studies have shown a process of biotic homogenization at a regional scale in the northeastern Brazilian Atlantic Forest (Lôbo et al. 2011), here we are expanding this evidence across multiple landscapes in the whole biome (but see de Lima, de Oliveira, et al. 2020 for evidences at a fragment scale), suggesting habitat fragmentation as a driver of biotic homogenization in tree communities across landscapes in the Brazilian Atlantic Forest.

Regarding endemism, our results report the effect of habitat cover and temperature seasonality as the strongest predictors affecting positively the endemism level. The positive relationship between habitat amount and endemism level aligns with the species‐area relationship, which suggests that larger areas (in our case landscapes with more habitat cover), tend to harbor more species, including endemics, by reducing the extinction risk (Storch et al. 2012; Hobohm et al. 2019). However, we highlight that in the highly fragmented Atlantic Forest, each forest remnant regardless of its size has an immense value to biodiversity conservation (Riva and Fahrig 2022; Vancine et al. 2024). This underscores the importance of a conservation strategy that integrates both large and small habitat fragments, following the approach of single large and several small (SLASS) (Szangolies et al. 2022), which may be more effective in preserving the Atlantic Forest biodiversity rather than choosing a unique type of conservation, such as the single large or several small (SLOSS) approach (Fahrig et al. 2022). Maintaining larger habitat fragments while simultaneously preserving smaller patches that function as stepping stones could enhance species persistence in the landscape, especially for endemics with limited dispersal abilities, by diminishing the mean spatial distance between communities. As we saw in our study, shorter distances help to sustain higher beta diversity across landscapes in the Brazilian Atlantic Forest.

Our study highlights the complex interplay of habitat fragmentation, habitat amount, and spatial distance in shaping beta diversity and endemism of tree communities across landscapes in the Brazilian Atlantic Forest. We demonstrated that spatial distance and landscape variables have a stronger influence as predictors of biodiversity patterns (beta diversity and endemism) compared to regional climate when evaluating replicated landscapes across a large geographic region. Moreover, our study successfully disentangles the effects of habitat fragmentation from those of habitat amount along a full gradient of habitat cover, which allows for a clearer understanding of how these two processes independently affect beta diversity. The historical context of the Atlantic Forest, which has experienced extensive habitat loss and fragmentation since before the 20th century (de Lima, de Oliveira, et al. 2020; Solórzano et al. 2021), further underscores the ongoing process of biotic homogenization in highly fragmented landscapes that we are suggesting here. Future studies should integrate additional factors, such as the time since fragmentation or temporal changes in habitat configuration for a given landscape area to provide a more comprehensive view of how fragmentation influences biodiversity over time (e.g., Collins et al. 2017). Although most landscapes in our dataset contained only two forest inventories, this sampling design is statistically adequate to estimate beta diversity and allows robust evaluation of fragmentation effects across the Brazilian Atlantic Forest; however, a greater number of inventories per landscape would likely capture intra‐landscape heterogeneity with higher precision. Finally, we advocate for conservation strategies that protect both large and small habitat patches to sustain biodiversity in human‐modified landscapes.

## Author Contributions

**Jean M. Freitag Kramer:** conceptualization, data curation, formal analysis, investigation, methodology, visualization, writing – original draft, writing – review and editing. **Victor P. Zwiener:** conceptualization, data curation, investigation, methodology, visualization, supervision, writing – original draft, writing – review and editing. **Mateus Camana:** methodology, visualization, writing – original draft, writing – review and editing. **Renato A. F. de Lima:** data curation, funding acquisition, investigation, methodology, writing – review and editing. **Sandra Cristina Müller:** conceptualization, funding acquisition, investigation, methodology, supervision, visualization, writing – original draft, writing – review and editing.

## Funding

This work was supported by Coordenação de Aperfeiçoamento de Pessoal de Nível Superior (Grant 001), Fundação de Amparo à Pesquisa do Estado de São Paulo (Grant 13/08722‐5), and Conselho Nacional de Desenvolvimento Científico e Tecnológico (Grants 314309/2023‐3 and 350132/2025‐9).

## Conflicts of Interest

The authors declare no conflicts of interest.

## Supporting information


**Appendix S1:** gcb70855‐sup‐0001‐supinfo.docx.

## Data Availability

Raw data on species abundance and endemism used in this study are stored in the Neotropical Tree Communities (TreeCo) database (de Lima et al. 2015; de Lima, de Oliveira, et al. 2020) and are available upon request at http://labtrop.ib.usp.br/doku.php?id=projetos:treeco:start. The processed data and R code that support the findings are available in the Zenodo repository at https://doi.org/10.5281/zenodo.16761037 (Kramer et al. 2025).
