# Supplementary material for: Effects of Deforestation and Fragmentation on Tree Beta Diversity and Endemism Across Landscapes in the Atlantic Forest Biodiversity Hotspot
Source: Glob Chang Biol. 2026 Apr 9;32(4):e70855. doi: 10.1111/gcb.70855 (PMC13066765; doi:10.1111/gcb.70855)
Supplement: Supplementary file 1 — Appendix S1: gcb70855‐sup‐0001‐supinfo.docx. [file GCB-32-e70855-s001.docx]

**Supporting information**

**Effects of deforestation and fragmentation on tree beta diversity and endemism across landscapes in the Atlantic Forest biodiversity hotspot**

Jean M. Freitag Kramer, Victor P. Zwiener, Mateus Camana, Renato A. F. de Lima, Sandra Cristina Müller


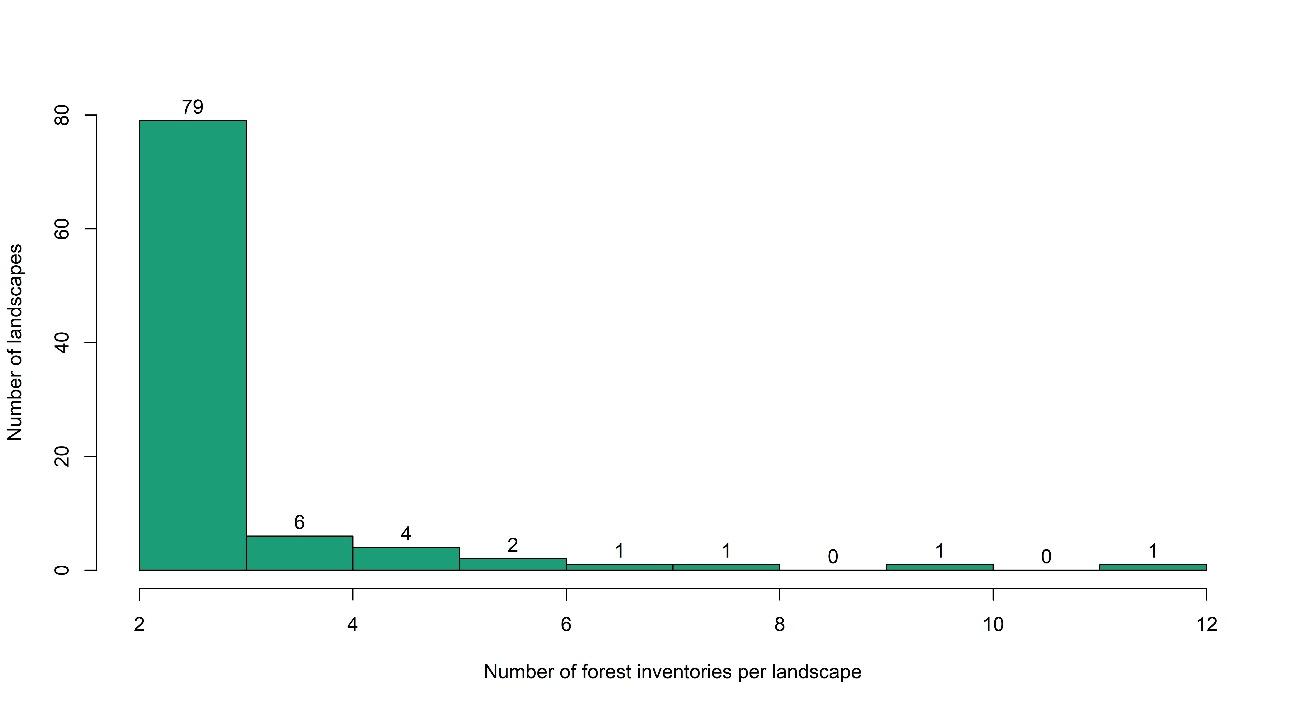
**Appendix S1.** Number of forest inventories per landscape considering our 95 studied landscapes in the Brazilian Atlantic Forest. The mean number of forest inventories per landscape was 2.84, with a median of 2. For instance, of the 95 studied landscapes, 79 (83%) had two forest inventories.


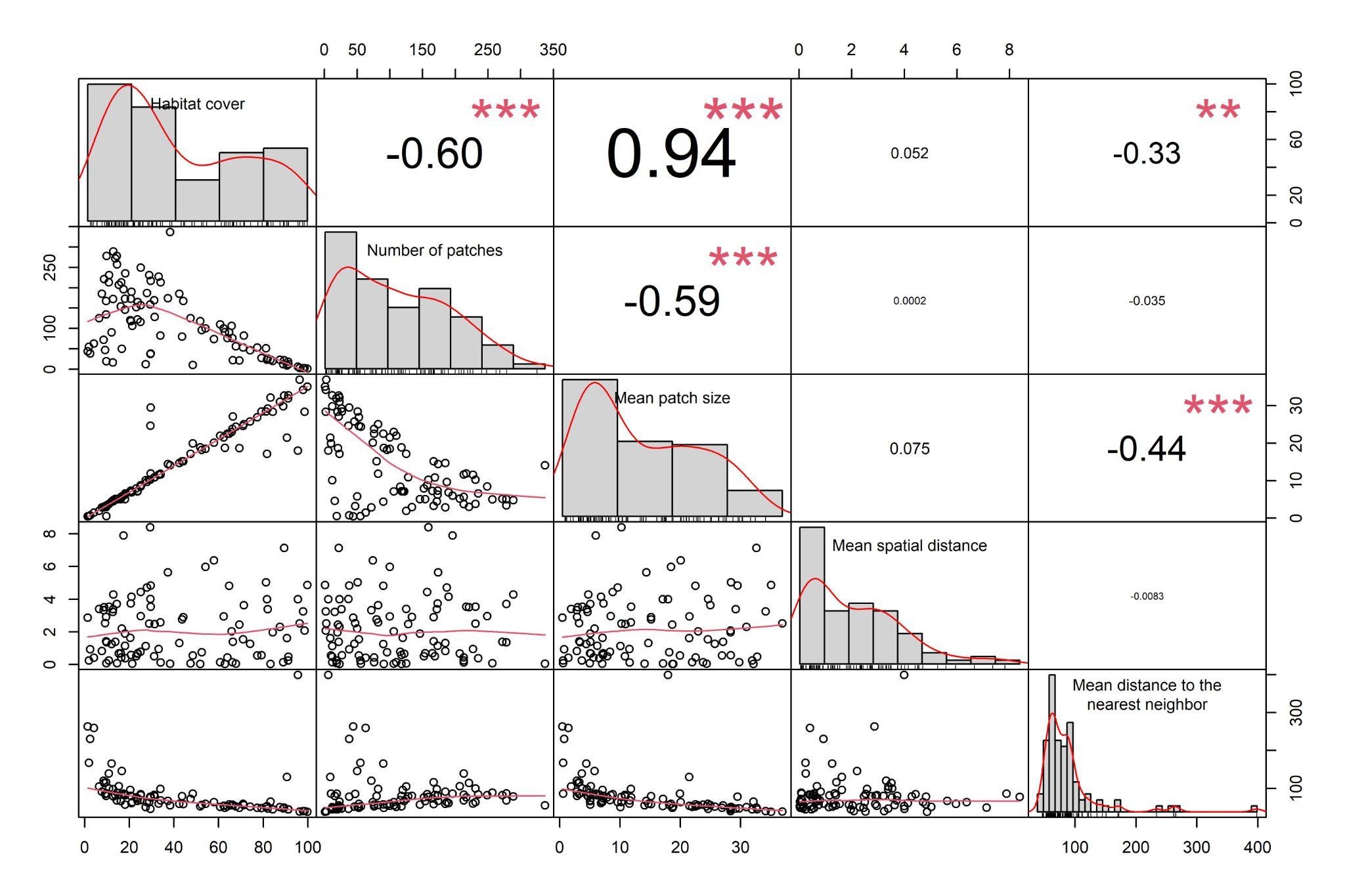


**Appendix S2.** Scatterplots comparing the relationships and Pearson correlation coefficients between the landscape variables (habitat cover, number of patches, mean patch size, mean spatial distance, and mean distance to the nearest neighbor) extracted for each landscape across the Brazilian Atlantic Forest. The upper/right panels contain the Pearson correlation coefficient, and the lower/left panels show pairwise scatterplots between each variable. The font size of the correlation coefficient is proportional to its value. Note that we excluded mean patch size from the analyses given its high correlation with habitat cover (r = 0.94).


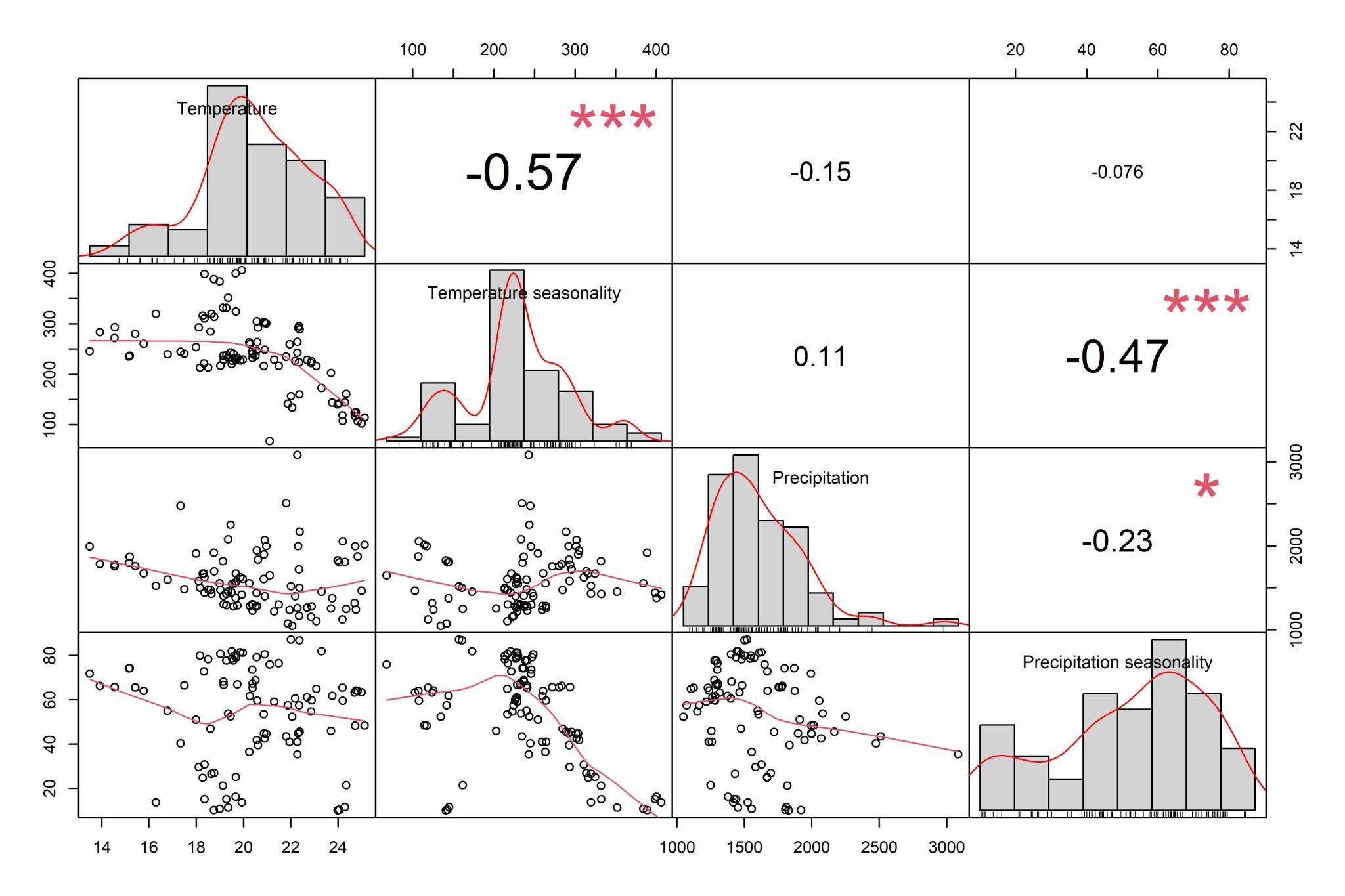


**Appendix S3.** Scatterplots comparing the relationships and Pearson correlation coefficients between the climate variables (temperature, temperature seasonality, precipitation, and precipitation seasonality) extracted within each landscape across the Brazilian Atlantic Forest. The upper/right panels contain the Pearson correlation coefficient, and the lower/left panels show pairwise scatterplots between each variable. The font size of the correlation coefficient is proportional to its value.


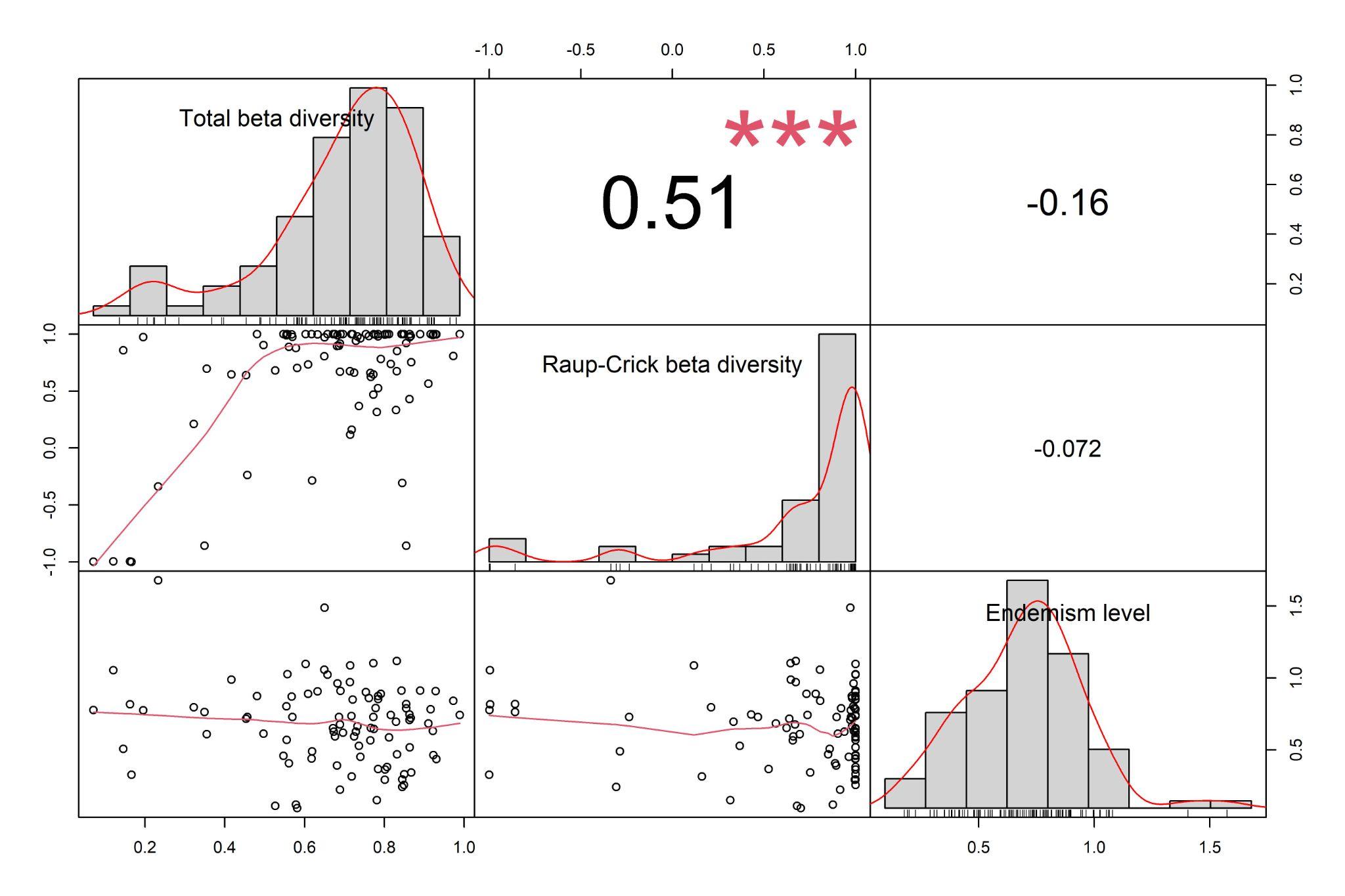


**Appendix S4.** Scatterplots comparing the relationships and Pearson correlation coefficients between the response variables (total beta diversity, Raup-Crick beta diversity, and endemism level) of tree communities in each landscape across the Brazilian Atlantic Forest. The upper/right panels contain the Pearson correlation coefficient, and the lower/left panels show pairwise scatterplots between each variable. The font size of the correlation coefficient is proportional to its value.


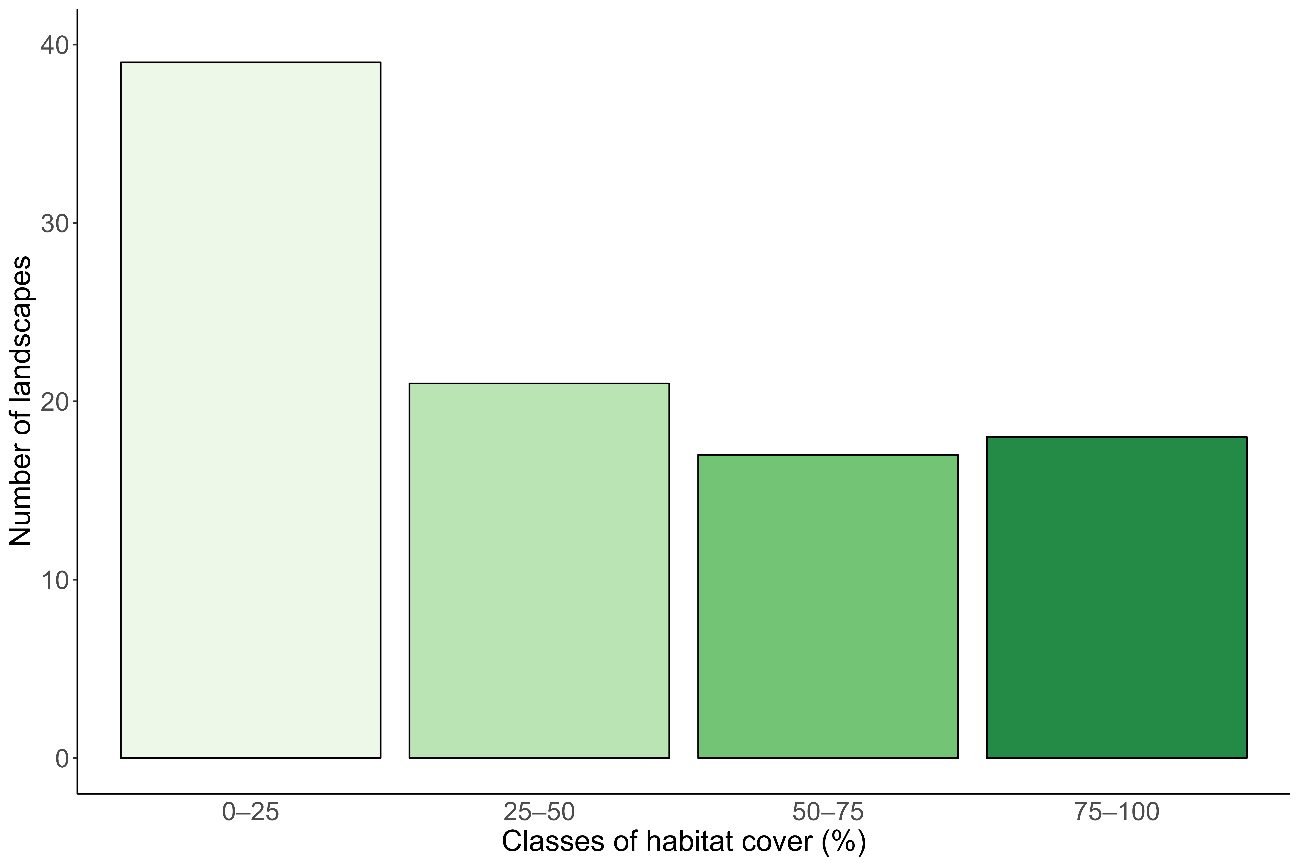


**Appendix S5.** The distribution of the 95 studied landscapes along the Brazilian Atlantic Forest representing the entire gradient of habitat amount, ranging from ~0 to ~100% of habitat cover. The habitat amount was divided into classes of habitat cover for a better representation.

**Appendix S6.** Loadings of principal component analysis (PCA) characterizing the environmental space across the 95 studied landscapes in the Brazilian Atlantic Forest. The axis with the highest loading for each variable is highlighted in bold.

| **Variable** | **PCA1 (30.89%)** | **PCA2 (19.18%)** | **PCA3 (15.30%)** |
| --- | --- | --- | --- |
| Habitat cover | **-0.527** | 0.208 | -0.224 |
| Number of patches | 0.387 | **-0.453** | -0.211 |
| Mean distance to the nearest neighbor | 0.266 | 0.085 | **0.418** |
| Mean spatial distance | 0.049 | 0.157 | **-0.488** |
| Temperature | 0.240 | **0.595** | 0.306 |
| Temperature seasonality | -0.314 | **-0.582** | 0.275 |
| Precipitation | **-0.435** | 0.156 | -0.215 |
| Precipitation seasonality | 0.389 | -0.008 | **-0.524** |

**Appendix S7.** Best beta regression models for Raup-Crick beta diversity following AIC statistics. All models with ΔAIC ≤ 2 are exhibited and ranked based on their AIC. Asterisks indicate variables that had significant effects (p < 0.05). Abbreviations: models = models name; AIC = Akaike information criteria; ΔAIC = AIC difference from the best model; R² = explained variance by the model; explanatory variables = predictors variables included in the model; Effect = standardized effect size; p = significance p value.

| **Models** | **AIC** | **ΔAIC** | **R²** | **Explanatory variables** | **Effect** | **p** |
| --- | --- | --- | --- | --- | --- | --- |
| Model 1 | -408.43 | 0 | 0.15 | Mean spatial distance | 0.31 | 0.007* |
|  |  |  |  | Number of patches | -0.18 | 0.13 |
| Model 2 | -408.30 | 0.13 | 0.12 | Mean spatial distance | 0.31 | 0.009* |
| Model 3 | -406.82 | 1.60 | 0.13 | Mean spatial distance | 0.31 | 0.009* |
|  |  |  |  | Mean distance to the nearest neighbor | 0.09 | 0.43 |
| Model 4 | -406.73 | 1.70 | 0.17 | Mean spatial distance | 0.31 | 0.008* |
|  |  |  |  | Number of patches | -0.17 | 0.14 |
|  |  |  |  | Mean distance to the nearest neighbor | 0.08 | 0.48 |
| Model 5 | -406.57 | 1.85 | 0.16 | Mean spatial distance | 0.32 | 0.007* |
|  |  |  |  | Number of patches | -0.19 | 0.11 |
|  |  |  |  | Temperature | -0.07 | 0.54 |
| Model 6 | -406.52 | 1.90 | 0.12 | Mean spatial distance | 0.31 | 0.008* |
|  |  |  |  | Precipitation | 0.07 | 0.52 |
| Model 7 | -406.47 | 1.95 | 0.12 | Mean spatial distance | 0.31 | 0.009* |
|  |  |  |  | Habitat cover | 0.07 | 0.55 |


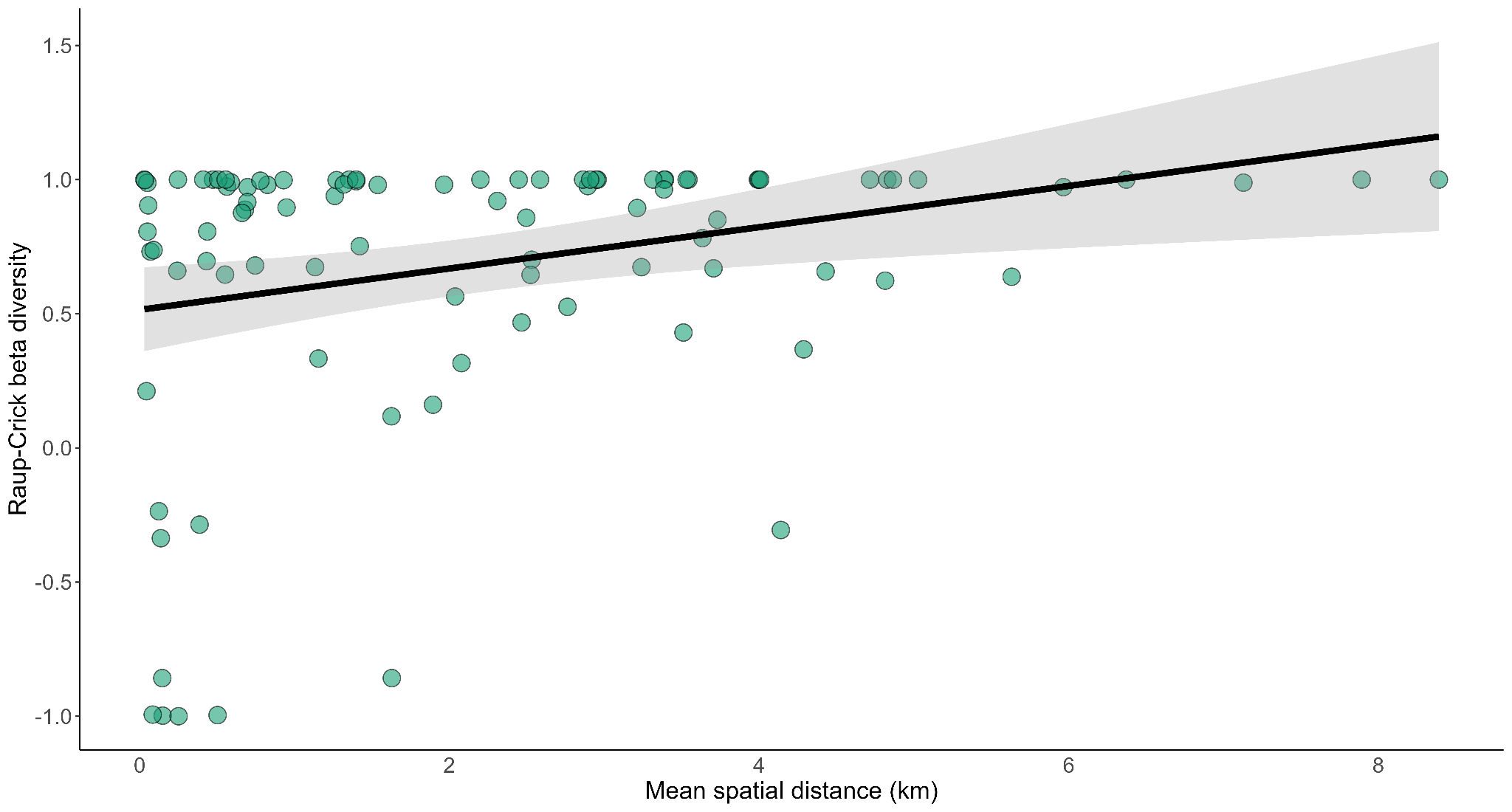


**Appendix S8.** Mean spatial distance was the strongest predictor which affects Raup-Crick beta diversity positively. Solid lines represent the relationships between variables and shaded gray areas represent the confidence intervals. The green points represent the landscapes along the Brazilian Atlantic Forest.
